# The expression and clinical significance of programmed cell death receptor 1 and its ligand in tumor tissues of patients with extranodal nasal NK/T cell lymphoma

**DOI:** 10.1038/s41598-021-02515-5

**Published:** 2022-01-07

**Authors:** Yu Feng, Xia Feng, Caixia Jing, Xinmei Yu, Yuhuan Zheng, Caigang Xu

**Affiliations:** 1 Department of Hematology/Hematology Research Laboratory, West China Hospital, Sichuan University, #37 Guo Xue Xiang Street, Chengdu, 610041 China; 2 Department of Traditional Chinese Medicine, The Public Health Clinical Center of Chengdu, Chengdu, China

**Keywords:** Cancer microenvironment, Cancer, Non-hodgkin lymphoma

## Abstract

Appropriate biomarkers may help distinguish the biological behavior of different types of lymphoma and their response to traditional chemotherapy. Extranodal natural killer/T-cell lymphoma (ENKTL) and diffuse large B-cell lymphoma (DLBCL) belong to different subtypes of non-Hodgkin's lymphoma, the biological behavior and prognosis of them are very different, programmed cell death receptor 1 (PD-1) and its ligand (PD-L1) have been investigated in these two types of diseases. However, few studies addressed the difference of PD-1/PD-L1 levels between ENKTL and DLBCL, in order to find out the difference and related clinical application value, the clinical data and tumor tissue paraffin sections of 24 newly diagnosed ENKTL patients and 42 newly diagnosed diffuse large B-cell lymphoma (DLBCL) were collected. PD-1/PD-L1 levels in tumor tissues were detected by immunohistochemical staining. The relationship between the PD-1/PD-L1 levels and clinical data of patients with ENKTL patients was analyzed. Both patient groups showed PD-1 level in tumor tissue of ENKTL patients was significantly lower than that of DLBCL patients (P < 0.05), while the PD-L1 level in tumor tissues of ENKTL patients was not different from DLBCL (P < 0.05). In addition, the ENKTL patients with B symptoms, elevated lactate dehydrogenase (LDH) levels and decreased hemoglobin (HGB) concentrations had lower level of PD-1 in tumor tissue. PD-L1 level in tumor tissues, the LDH level, Epstein-Barr genome (EBV-DNA) copy and Ki-67 index may affect the outcomes of ENKTL patients (P < 0.05), but they were not independent factors. PD-L1 levels in tumor tissues has clinical significance in ENKTL patients, which suggested that the PD-1/PD-L1 signal pathway may be involved in the immune escape of ENKTL and play different roles in different lymphoma subtypes.

## Introduction

Extranodal NK/T-cell lymphoma, nasal type (ENKTL) is an aggressive non-Hodgkin lymphoma which originates from T cell or NK-like cell, it often involve the nasal cavity, face and upper respiratory tract. Compared to diffuse large B-cell lymphoma (DLBCL), this disease is characterized by more difficult diagnosis, higher malignant, rapider progress, and easier to be resistant to traditional chemotherapy. Therefore, there is currently no standard treatment plan for ENKTL^1^. The above characteristics are probably related to tumor cells evading the immune response of the body, the signaling pathway involving programmed cell death receptor 1 (PD-1) is likely one mechanisms of immune evasion^2,3^.

Existing studies have shown that PD-1 is mainly expressed on CD4+ or CD8+ T cell membranes, and it has two types of ligands, which are PD-L1 and PD-L2. PD-L1 is the main ligand of PD-1 in the human body, PD-L1 is expressed in not only immune cells such as activated lymphocytes, NK cells and macrophages, but also tissue cells such as lung and vascular endothelium^2^. What is more, it is expressed at high levels in tumor cells, such as melanoma^4^, gastric cancer^5^, kidney cancer^6^, high levels of PD-L1 expressed on tumor cells can inhibit the proliferation and activation of lymphocytes by interacting with PD-1 on lymphocytes to achieve the immune escape of tumor cells^2^. Recent studies have shown that PD-L1 level in nasal biopsy tissues of ENKTL patients is much higher than that of rhinitis patients^7^. At present, the clinical trials of PD-1 monoclonal antibody are already underway. PD-1 monoclonal antibody has achieved very good outcomes in ENKTL^8^, but it didn’t achieve good outcomes in DLBCL^9^. Therefore, we hope to explore the reasons by comparing the expression differences of PD-1/PD-L1 level in these two diseases. Based on the above background, we used a retrospective case–control study to analyze the differences in PD-1/PD-L1 levels in tumor tissues of between ENKTL and DLBCL patients.

Therefore we examined PD-1/PD-L1 in tumor tissues of ENKTL and DLBCL patients. Our results show that PD-1/PD-L1 level in tumor tissues of ENKTL patients are useful for staging and prediction of treatment response in ENKTL. The same results were not obtained for patients with DLBCL, another type of lymphoma, suggesting that the biomarker may show some clinical significance for ENKTL.

## Materials and methods

### Study population and treatment

This retrospective study included 24 cases of ENKTL (18 males, median age 49.5 years) diagnosed in West China Hospital of Sichuan University from December 2017 to December 2018 and 42 cases of DLBCL patients, and pathological paraffin sections of all 66 patients have been obtained from the Department of Pathology, West China Hospital, Sichuan University. The diagnostic criteria are based on the 2008 WHO classification criteria for hematopoietic and lymphoid tissue tumors^10^. The outcomes evaluation after two courses of chemotherapy follows the "Evaluation Criteria for the outcomes of Malignant Lymphoma", including complete remission (CR), partial remission (PR), stable disease (SD) and disease progression (PD)^11^. We have signed an informed consent form with all research subjects, and have been approved by the Ethics Committee of West China Hospital of Sichuan University. All methods are performed in accordance with the relevant guidelines and regulations.

### Immunohistochemical staining (IHC) for PD-1/PD-L1

Paraffin-embedded sections (3 µm) were used for immunohistochemical staining (IHC). After being dewaxed and hydrated, antigen retrieval (citric acid with PH = 9) and inactivation of endogenous peroxidase, the primary antibody [PD-L1 antibody [28-8] (Abcam, Cambridge, UK)/PD-1 antibody (Zhongshan Jinqiao, Beijing, China), dilution 1:75] and general secondary antibody for antibody incubation, after washing with running water, DAB is added dropwise to develop color, after the color is developed, hematoxylin counterstaining is carried out, and then dehydrated and mounted. Observe the slices under a microscope and collect images, IHC was accomplished by avidin–biotin peroxidase complex detection system. The percentage of PD-1/PD-L1 expression (the number of cells stained with membranes in the total number of tumor cells) was analyzed by Image J software. The percentage > 30% was defined as PD-L1 (+), while the percentage > 5% was considered PD-1 (+)^12^.

### Statistical analyses

PD-1/PD-L1 level in tumor tissues are presented as the median and interquartile range [M(IQR)]. The Kruskal–Wallis test was used to analyze the differences between groups, and the Mann–Whitney U test and Spearman rank correlation analysis were used to analyze the relationship between PD-1/PD-L1 level in tumor and the clinical data of patients. Fish test and Logistic regression were used to conduct univariate and multivariate analysis of predictors of treatment response in ENKTL patients. We set P value < 0.05 as the significant difference.

### Ethics approval and consent to participate

This study was approved by the Ethics Committee of West China Hospital of Sichuan University. Participants provided written consent for their anonymized clinical data to be used and published for research purposes.

## Results

### Baseline clinical characteristics of patients

Baseline clinical characteristics of 24 patients with ENKTL and 42 ones with DLBCL are summarized in Table 1. In the ENKTL group, 7 (29%) was in stage III-IV, and 8 (33%) had LDH > 250 U/L. 23 (88%) patients accepted anthracycline-containing chemotherapy regimens. All patients received at least two cycles of chemotherapy before radiotherapy. After two courses of chemotherapy, 8 patients (33%) achieved CR.

**Table 1:** Baseline and clinical characteristics of 66 patients.

Characteristics	ENKTL patients (n = 24)	DLBCL patients (n = 42)
**Age, year**		
> 60	5 (21)	18 (43)
≤ 60	19 (79)	24 (57)
**Sex**		
Male	18 (75)	19 (45)
Female	6 (25)	23 (55)
**Ann Arbor stage**		
I–II	17 (71)	29 (69)
III–IV	7 (29)	13 (31)
**Bulky mass**		
Yes	1 (4)	11 (26)
No	23 (96)	31 (74)
**B symptoms**		
Yes	14 (58)	15 (36)
No	10 (42)	27 (64)
**Extranodal sites**		
> 1	20 (83)	8 (19)
≤ 1	4 (17)	34 (81)
**ECOG PS**		
≥ 1	4 (17)	12 (29)
< 1	20 (83)	32 (71)
**Bone marrow involvement**		
Yes	1 (4)	2 (5)
No	23 (96)	40 (95)
**Lactate dehydrogenase (U/L)**		
> 250	8 (33)	17 (40)
≤ 250	16 (67)	25 (60)
**EBER(+)**		
Yes	24 (100)	–
No	0 (0)	–
**PINK-E and IPI**		
Low (0–2)	16 (67)	33 (79)
High (≥ 3)	8 (33)	9 (21)
**Chemotherapy**		
Anthracycline	21 (88)	–
Non-anthracyline	3 (12)	–
**Response after two treatment courses**		
Complete remission	8 (33)	–
Other	16 (67)	–

Values are n (%).

*DLBCL* diffuse large B-cell lymphoma, *ECOG PS* Eastern Cooperative Oncology Group performance score, *ENKTL* extranodal natural killer/T-cell lymphoma, *PINK-E* prognostic index of extranodal natural killer/T-cell lymphoma, *IPI* international prognostic index, “–” data not collected.

### PD-1 and PD-L1 levels in tumor tissues and relationships with clinical variables

**Figure 1:**
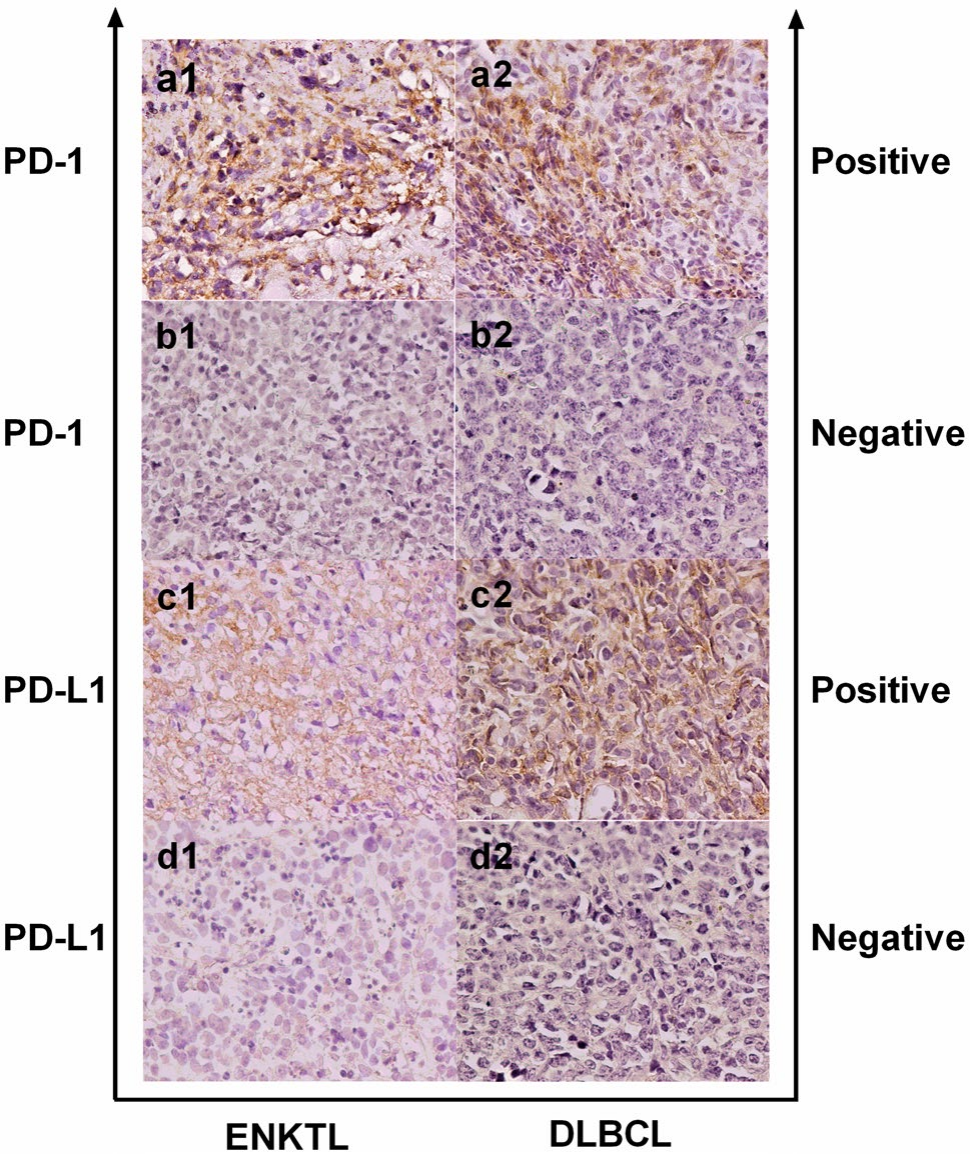
Immunohistochemical staining of PD-1 (a1,a2,b1,b2) and PD-L1 (c1,c2, d1,d2) under the microscope of ENKTL and DLBCL.

**Figure 2:**
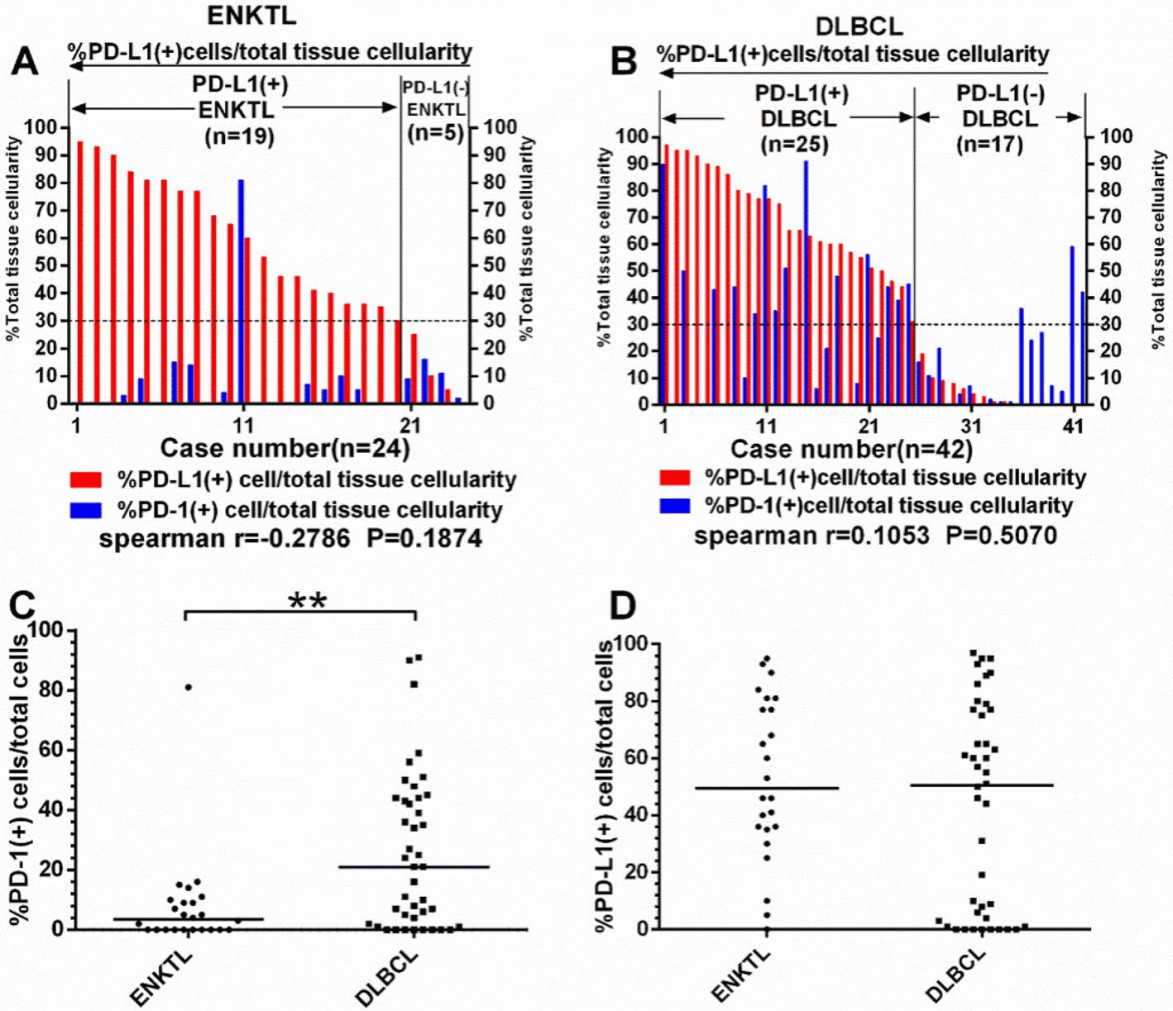
Details and correlation analysis of PD-1/PD-L1 expression in tumor tissues of ENKTL (**A**) and DLBCL (**B**) patients. No correlation was found between PD-1 and PD-L1 level from tumor tissues of ENKTL and DLBCL patients. Scatter plot of PD-1 (**C**) and PD-L1 (**D**) expression showed that PD-1 level in tumor tissues from ENKTL patients was lower than DLBCL.**P < 0.01.

**Figure 3:**
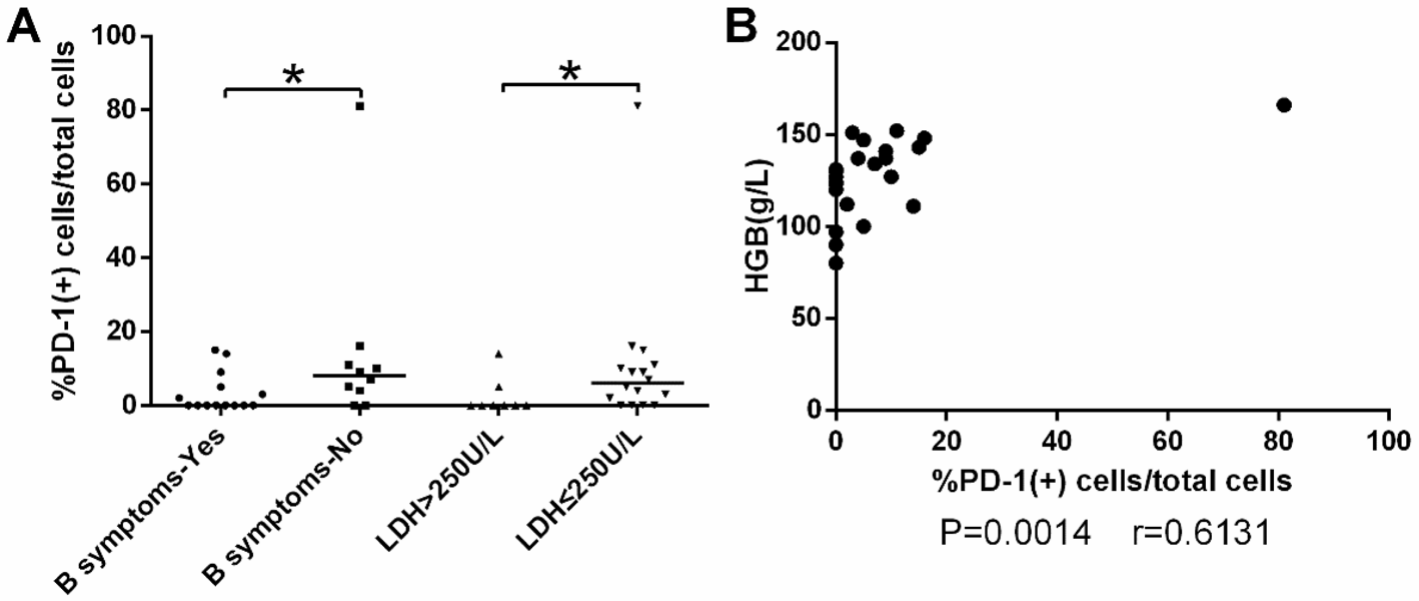
(**A**,**B**) In ENKTL patients, PD-1 level in tumor tissues varied with (**A**) B symptoms^**#**^, (**A**) lactate dehydrogenase (LDH) concentration, (**B**) hemoglobin. *P < 0.05. ^**#**^The B symptoms of lymphoma mainly refer to systemic symptoms, which usually include three aspects: (1) Fever, with a body temperature above 38 °C, usually for more than 3 consecutive days, but there is no clear clinical cause of infection. (2) The weight loss reached more than 10% within 6 months. (3) Night sweats. The so-called night sweats refer to the sweating of the patient after falling asleep.

The distribution and expression of PD-1/PD-L1 in tumor tissues were investigated by immunohistochemical staining. Membranes of positive cells in tumor tissues from ENKTL and DLBCL patients were stained brown (Fig. 1). PD-L1 (+) was defined as positive cells proportion > 30% and PD-1 (+) as > 5%^12^. In 24 ENKTL patients, 19 (79%) were PD-L1 (+) and 9 (38%) were PD-1(+) (Fig. 2A). In 42 DLBCL patients, 25 (60%) were PD-L1 (+) and 29 (69%) were PD-1 (+) (Fig. 2B). PD-1 level in tumor tissues in ENKTL patients [3.50(0.00–9.75)] was significantly lower than in DLBCL patients [21.00(1.75–44.00), P = 0.0021] (Fig. 2C), while PD-L1 level was not statistically different between ENKTL [49.50(35.25–80.00)] and DLBCL [50.50(2.50–77.00)] groups (Fig. 2D). In ENKTL patients, PD-1 level in tumor tissues were significantly lower with B symptoms^**#**^, higher levels of LDH (P < 0.05, Fig. 3A), and lower hemoglobin level (Spearman r = 0.6131, P = 0.0014, Fig. 3B).

### Association of PD-1 or PD-L1 biomarkers with treatment response

As shown in Table 2, Univariate analysis showed that ENKTL patients with higher levels of PD-L1 in tumor tissues (P = 0.0218) were more difficult to achieve CR, as were patients with high levels of LDH (P = 0.0222), Ki-67 index (P = 0.0304) and EBV-DNA copies (P = 0.0087). In multivariate analysis, the covariates we chose are LDH, EBV-DNA copies, Ki-67 index, PD-L1 level in tumor tissues, however, no one varied significantly with treatment response.

**Table 2:** Univariate and multivariate analyses to identify predictors of treatment response among ENKTL patients.

Variable	Univariate analysis		P	Multivariate analysis		P
	Complete remission (n = 8)	Other treatment response (n = 16)		OR	95% CI	
**Age > 60 (years)**			0.2885			
Yes	3 (38)	2 (14)				
No	5 (62)	14 (86)				
**Sex**			0.3618			
Male	5 (62)	13 (81)				
Female	3 (38)	3 (19)				
**Ann Arbor stage**			0.3521			
I–II	7 (88)	10 (63)				
III–IV	1 (12)	6 (37)				
**Bulky mass**			1.000			
Yes	0 (0)	1 (6)				
No	8 (100)	15 (94)				
**B symptoms**			0.6734			
Yes	4 (50)	10 (63)				
No	4 (50)	6 (37)				
**Extranodal sites**			0.5784			
> 1	6 (75)	14 (86)				
≤ 1	2 (25)	2 (14)				
**ECOG PS**			0.5784			
≥ 1	2 (25)	2 (14)				
< 1	6 (75)	14 (86)				
**Bone marrow involvement**			0.3333			
Yes	1 (12)	0 (0)				
No	7 (88)	16 (100)				
**Lactate dehydrogenase > 250U/L**			0.0222	0.972	0.932–1.014	0.187
Yes	0 (0)	8 (50)				
No	8 (100)	8 (50)				
**PINK-E**			0.1893			
Low (0–2)	7 (88)	9 (56)				
High (≥ 3)	1 (12)	7 (44)				
**Chemotherapy**			0.2490			
Anthracycline	2 (25)	1 (6)				
Non-anthracyline	6 (75)	15 (94)				
Hemoglobin, g/L	135.5 (114–142.5)	127.0 (114–144.5)	0.6205			
Platelet count, 10^9^ /L	201.0 (164.5–235.8)	206.0 (172.8–296.5)	0.4435			
Lymphocyte count, 10^9^/L	1.435 (1.208–1.848)	1.290 (0.805–1.580)	0.4254			
Lymphocyte percentage, %	28.60% (18.90–31.05%)	22.05% (13.65–26.30%)	0.1199			
Copies of Epstein–Barr virus genome	32.70 (10.30–317.3)	1420 (155.3–35,025)	0.0087	0.994	0.840–1.022	0.171
Ki-67, %	47.50% (41.25–55.00%)	67.50% (46.25–73.75%)	0.0304	0.871	0.758–1.002	0.054
PD-1 level in tumor tissues, %	6.00% (2.50–10.50%)	0.00% (0.00–9.75%)	0.2256			
PD-L1 level in tumor tissues, %	35.50% (10.00–59.00%)	64.00% (41.50–83.25%)	0.0218	0.926	0.840–1.022	0.126

Values are n (%) or median (interquartile range).

*ENKTL* extranodal natural killer/T-cell lymphoma, *ECOG PS* Eastern Cooperative Oncology Group performance score, *PINK-E* Prognostic Index of extranodal natural killer/T-cell lymphoma, *OR* odd ratio, *95% CI*, 95%.

### Correlation among PD-1/PD-L1 level in tumor tissues, PD-L1mRNA level in PBMCs and sPD-L1 level

**Figure 4:**
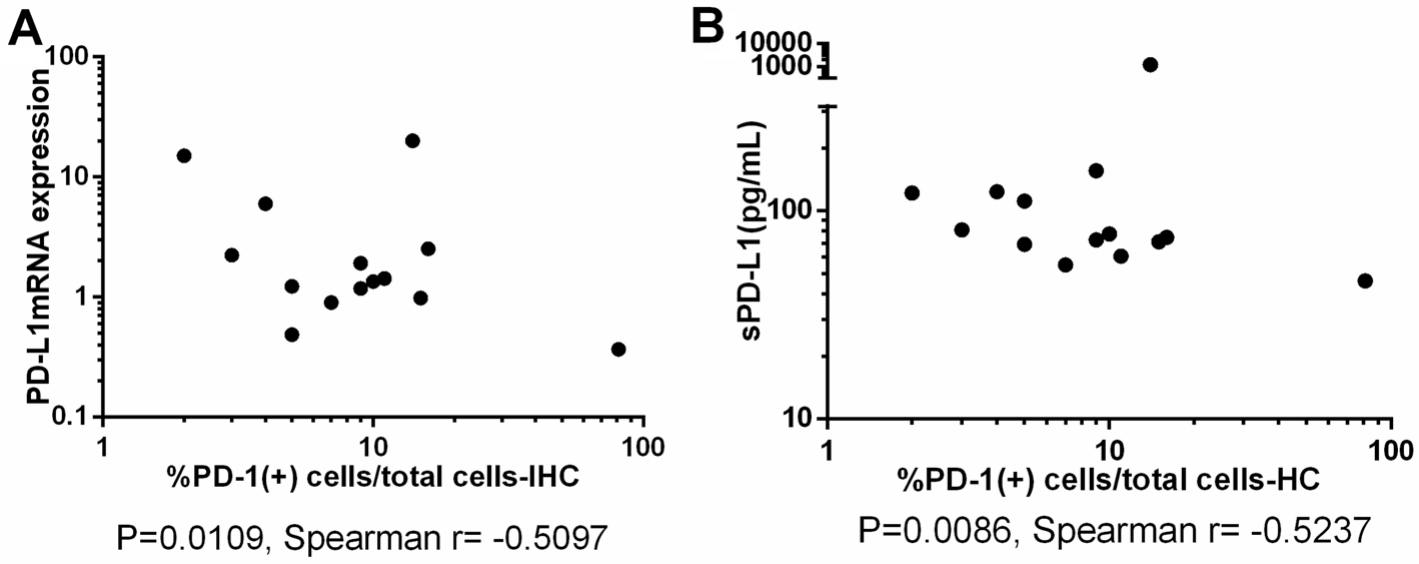
(**A**) Correlation between PD-1 level in tumor tissues and PD-L1mRNA level in PBMC. (**B**) Correlation between PD-1 level in tumor tissues and sPD-L1 level.

Early research by our research group detected expressions of PD-1/PD-L1 in peripheral blood mononuclear cells (PBMCs) and plasma. We used the same cohort of patients as before, however, pathological specimens of some patients are not available, so the number of patients included in this article has decreased. We made a correlation analysis of PD-1/PD-L1 level between tumor tissue and peripheral blood, our results showed PD-1 level in tissues was correlated with PD-L1mRNA level in PBMCs (P = 0.0109, Spearman r = − 0.5097, Fig. 4A) and sPD-L1 level (P = 0.0086, Spearman r = − 0.5237, Fig. 4B), Unfortunately, we didn’t find a correlation between PD-L1 level in tumor tissues and soluble PD-L1 level in peripheral blood.

## Discussion

Studies have found that in patients with follicular lymphoma, PD-1 is mainly expressed on CD4+ T lymphocytes in lymph node follicles^13^. Roncador G, García Verdes-Montenegro JF found that PD-1 is abnormally highly expressed in tissues of patients with angioimmunoblastic lymphoma (AITL)^14^. Jo et al. have reported the low level of PD-1 in tumor tissues of ENKTL patients^15^, and Muhamad et al. also found that PD-1 was positive in 20.5% of stroma, but undetectable on lymphoma cells^16^. Similarly, this study found that PD-1 level in tumor tissues of ENKTL patients was significantly lower than that of DLBCL patients, and that PD-1 level in tumor tissues of ENKTL patients with B symptoms, increased LDH levels, and decreased hemoglobin concentration was lower. We believe that the low level of PD-1(+) infiltrating lymphocytes constitutes the immunosuppressive effect of lymphoma cells in tumor microenvironment and promotes tumor immune escape, from this perspective, the lower PD-1 level in ENKTL tumor tissue may be one of the possible reasons why ENKTL progresses faster than DLBCL.

In addition, Chen BJ, Chapuy B and other scholars have found that PD-L1 is expressed in HL, DLBCL, ENKTL and other lymphoma tissues, but no expression has been detected in Burkitt lymphoma^17^, similar to this, this study is also found that PD-L1 is highly expressed in tumor tissues of ENKTL and DLBCL patients, it is suggested that the increased expression of PD-L1 is one of the possible reasons for the defect of cellular immune function in patients.

The outcomes-related analysis in ENKTL patients found that the LDH level, EBV-DNA copy number, Ki-67 index and PD-L1 level in tumor tissues will affect the outcomes of ENKTL patients, but are not independent factors. Li et al. also found that Persistent peripheral blood EBV-DNA positive with high expression of PD-L1 and upregulation of CD4 + CD25 + T cell ratio in early stage NK/T cell lymphoma patients may predict worse outcome^18^. Similarly, we also found high levels of EBV-DNA copies were more difficult to achieve CR.

Bi et al. found that Patients with a high concentration of serum soluble PD-L1 or with a high percentage of PD-L1 expression in tumor specimens exhibited significantly lower response rate to treatment and remarkably worse survival, compared with their counterparts^19^. Wang et al. also found that Patients with high pretreatment had shorter progression-free survival and overall survival^20^. However, Kim found that PD-L1 expression was the only significant independent predictor for longer OS in patients with advanced stage (III/IV) ENKTL^21^. For this contradictory result, We analyzed the patient staging of three articles, We found that the patients studied in the first two articles are all classified as stage I–II patients, the third article was for patients with stage III–IV. We believed that PD-L1-mediated inhibition may lead to local depletion of cytokines involved in the survival and growth of cancer cells, resulting in anti-tumor effects in patients with advanced stage (III/IV) ENKTL, This also showed that PD-L1 played different roles in ENKTL patients of different stages.

These suggest that the PD-1/PD-L1 signaling pathway may be involved in the disease progression of ENKTL patients, and is closely related to the patient's response to traditional chemotherapy.

In summary, the results of this study showed that the expression of PD-1/PD-L1 in the tumor tissues of patients is different in ENKTL and DLBCL patients, suggesting that the PD-1/PD-L1 signaling pathway has different roles in different lymphoma subtypes.

## Data Availability

The datasets generated and analyzed in the current study are available from the corresponding author on reasonable request.
